# Case Report: Chronic Cavitatory Pulmonary Aspergillosis after COVID-19

**DOI:** 10.4269/ajtmh.21-0701

**Published:** 2021-11-24

**Authors:** Sheetal Chaurasia, Manjunath Thimmappa, Saurav Chowdhury

**Affiliations:** ^1^ Department of Pulmonary Medicine, Manipal Hospital Whitefield, Bangalore, India;; ^2^ Department of Critical Care Medicine, Manipal Hospital Whitefield, Bangalore, India

## Abstract

Chronic pulmonary aspergillosis can present in four distinct clinical syndromes, one of which is chronic cavitary pulmonary aspergillosis (CCPA). CCPA is generally associated with a mildly immunosuppressed state or, in immunocompetent patients, with structural lung damage. Severe acute respiratory syndrome coronavirus 2 (SARS-CoV-2) infection has been associated with reactivation of previous quiescent infections such as tuberculosis and invasive fungal infections, but CCPA in a patient with COVID-19 is rarely reported. Here we present the case of a 57-year-old man with CCPA associated with COVID-19 infection in whom latent aspergilloma was most likely activated after SARS-CoV-2 infection. The patient presented with severe COVID and, after initial response to treatment, started to deteriorate due to reactivation of latent aspergilloma to a more aggressive CCPA form. After confirmation of the diagnosis, the patient was initiated on treatment with voriconazole. He showed a good response to treatment with clinicoradiological response. This case also depicts one of the common causes of clinical deterioration in otherwise recovering COVID-19 patients.

## INTRODUCTION

Chronic pulmonary aspergillosi*s* (CPA) complicates other respiratory disorders, with the most common form being chronic cavitary pulmonary aspergillosis (CCPA).^1^ Bronchopulmonary aspergillosis tends to be contracted by the inhalation of mycotic spores of *Aspergillus* species. These spores are ubiquitous in the environment and do not usually cause illness in immunocompetent people or people with normal lungs.^2^ When there is an underlying structural lung disease and/or an immunocompromised status, these spores can lead to four distinct pulmonary syndromes.^2^

Chronic pulmonary aspergillosis is well described in patients with underlying parenchymal lung disease.^2^ There have been infrequent reports of aspergillomas and CCPA in patients with acute COVID-19 and also after recovery from COVID-19.^3^^,^^4^ Here we present the case of a 57-year-old man with CCPA associated with COVID-19 in whom latent aspergilloma was most likely activated after severe acute respiratory syndrome coronavirus 2 (SARS-CoV-2) infection. The man did not have a history of immune suppression and activation, and increase in the size of the fungal ball was demonstrated during the course of his hospital stay for treatment of COVID-19, necessitating antifungal treatment.

## CASE REPORT

A 57-year-old man presented with cough, fever, and breathing difficulty for 2 days. His past medical history was significant for previously treated pulmonary tuberculosis (TB) 20 years earlier.

Clinical examination showed a heart rate of 100/minute, respiratory rate of 24/minute, oxygen saturation of 84%, and temperature of 100°F. Chest examination showed bilateral crackles. Other systems were within normal limits.

**Figure 1.**
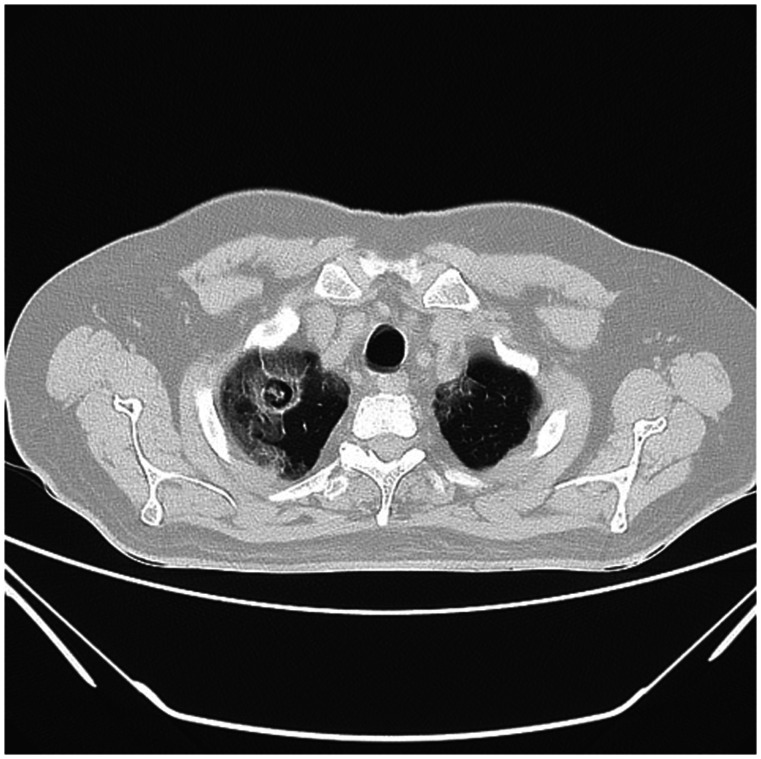
High-resolution computed tomography chest showed bilateral peripheral ground-glass opacities with a right upper lobe cavity measuring 2.3 × 2.3 cm containing an opacity inside which was likely a fungal ball.

Investigations showed a leukocytosis of 8,000 (4,000–11,000 cells/microliter), C-reactive protein (CRP) of 54 (< 10 mg/dL), lactate dehydrogenase (LDH) of 440 (140–280 U/L), ferritin of 800 (20–250 ng/mL), and a D-dimer of 548 (< 250 ng/mL). Liver function tests, renal function tests, and serum electrolytes were normal. COVID-19 reverse transcriptase polymerase chain reaction (RT-PCR) was positive. Chest x-ray showed bilateral interstitial infiltrates and a possible cavitatory lesion in the right upper zone. A high-resolution computed tomography (HRCT) chest showed bilateral peripheral ground-glass opacities with right upper lobe cavity measuring 2.3 × 2.3 cm containing an opacity inside which was likely a fungal ball (Figure 1). A provisional diagnosis of severe COVID-19 pneumonia with a coexisting aspergilloma was made, and he was admitted for treatment.

The patient was admitted to the intensive care unit and treated with intravenous dexamethasone, Remdisivir, Bipap, anticoagulation, and proning. He responded to treatment and was moved to the general ward on 6 L oxygen by face mask. However, on day 3 in the ward, he became increasingly hypoxic with oxygen requirement increased to 15 L/minute by non-rebreather mask.

**Figure 2.**
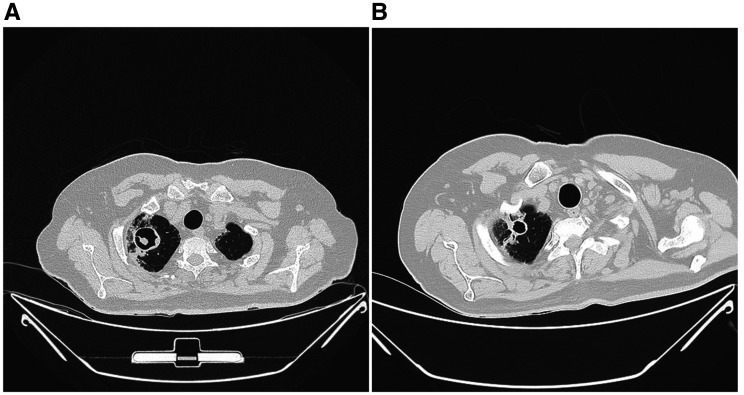
(**A**) High-resolution computed tomography (HRCT) thorax showing an increase in the size of the cavitatory lesion as well as the opacity inside it. (**B**) HRCT thorax showing the decreasing size of the cavitatory lesion with a resolved inner opacity.

Repeat laboratory investigation showed leukocytosis of 18,000 (4,000–10,000 cells/µL). However, the CRP, D-dimer, ferritin, and LDH showed a decreasing trend. The blood, sputum, and urine cultures were negative. Sputum was negative for acid-fast bacilli and GeneXpert. A repeat HRCT showed an increase in the right upper lobe cavity size to 3.3 × 3.2 cm with an increase in the soft tissue ball to 12 × 8 mm (Figure 2A). The cavity wall had become more irregular as well. The ground-glass opacities were resolving as compared with the initial HRCT. An increase in the size of the cavity and the opacity with associated irregularity of previously smooth cavity suggested possible invasion by the previously quiescent aspergilloma, likely becoming semiinvasive pulmonary aspergillosis.

An *Aspergillus*-specific immunoglobulin (Ig)G was positive. Serum galactomannan was 2 (< 0.5). On the basis of the clinical, radiological, and biochemical parameters, he was diagnosed to have chronic cavitating pulmonary aspergillosis. He was started on intravenous voriconazole 300 mg every 12 hours on day 1 followed by 200 mg every 12 hours subsequently, along with the continuation of the dexamethasone, after which the oxygen requirement was reduced and he could be weaned off oxygen and the steroid stopped. Repeat HRCT on day 23 of admission showed disappearance of the previously noted fungal ball, reduction in the size of the cavity, and thinning of the cavity wall (Figure 2B). The voriconazole was planned to continue orally to complete 6 weeks of treatment. He was discharged with a negative COVID-19 RT-PCR on this oral voriconazole. On review 2 months later, he was asymptomatic and was advised to continue the voriconazole for 2 more months.

## DISCUSSION

The diagnosis of CPA requires a combination of the following characteristics: a consistent appearance on thorax imaging, direct evidence of aspergillus infection or immunological response to *Aspergillus* spp., and exclusion of alternative diagnoses.^1^ Conventionally, CPA should be present for at least 3 months, although that duration may be inferred based on the patient’s symptoms or demonstrable progressive radiological abnormality.^1^ In our patient, although there was initially confusion concerning the exact terminology of the disease, the aspergilloma on the initial HRCT with raised *Aspergillus*-specific immunoglobulin (Ig)G as well as the reactivation and increase in size pointed to a chronic rather than an acute condition. His past pulmonary TB most likely resulted in a cavity, which facilitated the formation of an aspergilloma. Because he was immunocompetent, this aspergilloma likely remained quiescent until the infection with SARS-CoV-2. There have been reports of reactivation of TB after SARS-CoV-2 infection, suggesting that the complex immunological processes involved in this infection could lead to reactivation of dormant secondary infections.^5^ In a country such as India with a high burden of active and prior pulmonary TB and its sequalae, the reactivation of silent aspergilloma and progression to a more aggressive form of CPA remains a concern in this pandemic among severe COVID patients receiving high-dose steroids.

SARS-CoV-2–associated pulmonary aspergillosis (CAPA) has been described in COVID-19 patients with acute respiratory distress syndrome. The pathogenesis is incompletely understood but several immunological mechanisms may be responsible for the development of CAPA as well as other fungal infections.^6^ SARS-CoV-2 infection results in the release of danger-associated molecular patterns that act as endogenous signals with a subsequent immune and inflammatory response, resulting in lung injury. Another possibility involves the collateral effects of the host’s recognition pathways, which are required for the activation of antiviral immunity. These pathways, while protecting against viruses, may paradoxically contribute to an inflammatory environment that favors fungal pathogenesis.^6^

Despite being described in the literature, the diagnosis of CCPA and CAPA are not straightforward, especially in the setting of the current pandemic. In our patient, even though the cavity and the fungal opacity were found at the time of diagnosis of COVID-19, it was not treated because it was presumed to be an incidental finding without causing the patient any prior discomfort. It was only after the patient’s clinical condition deteriorated after initial recovery and a repeat thoracic imaging demonstrating an increase in the size of the cavitatory lesion that the possibility of invasive or chronic-invasive aspergillosis considered.

A minimum of 4 to 6 months of oral triazole therapy is recommended for the treatment of CCPA.^1^ In our patient, we started with intravenous voriconazole because he was acutely unwell at the time of initiation of therapy, followed by a switch to oral after he improved.

CCPA is rarely reported in COVID-19. Pulmonary aspergillosis has been reported in severe COVID-19 in varying degrees, but cavitation associated with *Aspergillus* infection is reported only in a handful of cases.^7^ This case is also important because the burden of tuberculosis is high in India. With a large number of cases with sequalae in the form of fibrocavity and aspergilloma. Such cases after contracting COVID-19 are at a higher risk of getting a more aggressive form of aspergillus infection

The diagnostic challenges associated with these cases include difficulty obtaining a microbiological diagnosis, heavy reliance on indirect markers such as immunological markers or radiological findings, and possible need for empirical antifungal therapy in the event of negative test results. The presence of elevated *Aspergillus*-specific IgG, however, can represent either active or prior infection.^8^ Its optimal cutoff level in different geographic areas also remains indeterminate.^9^ It has been found that baseline *Aspergillus*-specific IgG levels in an Asian population with intermediate TB burden can be expected.^10^ Therapeutic challenges include poor clinical outcomes associated with severe COVID-19, which is worsened by the progressive cavitation and lung damage.

Determinants of clinical outcome include general clinical status of the patient, comorbidities, stage at which the diagnosis of aspergillosis is confirmed, and host response to the treatment

We wish to highlight the possibility of superadded invasive fungal infections in seemingly immunocompetent individuals with COVID-19. SARS-CoV-2 infection and treatment with steroids could play a role in the reactivation of previously quiescent infections. In a patient with COVID-19 who deteriorates after initial improvement, it may be worthwhile to look for an underlying secondary infection, as was found in our case. Treatment of CCPA can lead to favorable outcomes in the setting of early recognition and prompt treatment of the condition.
